# New Insights into the Hepcidin-Ferroportin Axis and Iron Homeostasis in iPSC-Derived Cardiomyocytes from Friedreich's Ataxia Patient

**DOI:** 10.1155/2019/7623023

**Published:** 2019-03-27

**Authors:** Alessandra Bolotta, Provvidenza Maria Abruzzo, Vito Antonio Baldassarro, Alessandro Ghezzo, Katia Scotlandi, Marina Marini, Cinzia Zucchini

**Affiliations:** ^1^Department of Experimental, Diagnostic and Specialty Medicine, Bologna University, 40126 Bologna, Italy; ^2^IRCCS Fondazione Don Carlo Gnocchi, 20148 Milan, Italy; ^3^Interdepartmental Centre for Industrial Research in Health Sciences and Technologies (ICIR-HST), University of Bologna, 40064 Ozzano, Bologna, Italy; ^4^CRS Development of Biomolecular Therapies, Experimental Oncology Laboratory, Orthopedic Rizzoli Institute, 40136 Bologna, Italy

## Abstract

Iron homeostasis in the cardiac tissue as well as the involvement of the hepcidin-ferroportin (HAMP-FPN) axis in this process and in cardiac functionality are not fully understood. Imbalance of iron homeostasis occurs in several cardiac diseases, including iron-overload cardiomyopathies such as Friedreich's ataxia (FRDA, OMIM no. 229300), a hereditary neurodegenerative disorder. Exploiting the induced pluripotent stem cells (iPSCs) technology and the iPSC capacity to differentiate into specific cell types, we derived cardiomyocytes of a FRDA patient and of a healthy control subject in order to study the cardiac iron homeostasis and the HAMP-FPN axis. Both CTR and FRDA iPSCs-derived cardiomyocytes express cardiac differentiation markers; in addition, FRDA cardiomyocytes maintain the FRDA-like phenotype. We found that FRDA cardiomyocytes show an increase in the protein expression of HAMP and FPN. Moreover, immunofluorescence analysis revealed for the first time an unexpected nuclear localization of FPN in both CTR and FRDA cardiomyocytes. However, the amount of the nuclear FPN was less in FRDA cardiomyocytes than in controls. These and other data suggest that iron handling and the HAMP-FPN axis regulation in FRDA cardiac cells are hampered and that FPN may have new, still not fully understood, functions. These findings underline the complexity of the cardiac iron homeostasis.

## 1. Introduction

Iron is a trace metal essential for numerous biological processes. Its homeostasis is finely regulated, since both iron excess and deficiency are potential detrimental. In fact, iron excess favors the formation of oxygen radicals, while iron deficiency impairs enzyme functionality affecting oxygen metabolism. It has been demonstrated that the dysregulation of iron homeostasis is involved in different pathological conditions, including cancer, anemia, neurodegenerative disorders, and cardiac diseases [1]. Iron deficiency was found to occur in heart failure patients, independently of normal systemic iron concentration, causing morphological and functional mitochondrial alterations and consequently ATP depletion [2]. These dysfunctions, in turn, impair cardiac contractility and relaxation. Ironically, cardiomyopathy can be induced also by systemic iron overload, as in hereditary hemochromatosis (HH) and *β*-thalassemia, and by iron misdistribution in the cellular organelles, as in Friedreich's ataxia (FRDA) [3]. Iron excess causes an alteration of systolic and diastolic functions through the decrease of L-type channel activity, essential for the heart contraction. In addition, at the cellular level, iron misdistribution in cellular organelles, such as the mitochondria, can damage the cells through oxygen radical production. Cardiomyocytes, being endowed of poor antioxidant defenses, are more susceptible to reactive species of oxygen (ROS) damage via Fenton and Heiber-Weiss-typereactions [3, 4]. Iron homeostasis is regulated by several proteins involved in the iron uptake, transport, storage, and export. These proteins cooperate with ferrireductases, ferroxidases, and chaperones to regulate the cellular iron trafficking and to limit the unbound labile iron pool (LIP), potential source of ROS. Iron exists within heme molecules such as hemoglobin and cytochromes or in iron-sulfur cluster- (ISC-) containing proteins such as succinate dehydrogenase; moreover, nonheme/non-ISC iron-containing proteins are present in the cells [5]. Nonheme iron is transported into the cells by iron-binding proteins, such as transferrin. Cellular uptake of iron from transferrin is initiated by the binding of transferrin to transferrin receptor 1 (TFRC). TFRC is a transmembrane protein that assists iron uptake through receptor-mediated endocytosis of iron-loaded transferrin [5]. In addition, iron chaperones such as frataxin, a nuclear-encoded protein localized into the mitochondrial matrix, act as iron sensor and storage proteins as well as iron chaperons during cellular Fe-S cluster biosynthesis [6].

In iron homeostasis, a central regulatory mechanism is the binding of the hormone hepcidin (HAMP) to the iron exporter ferroportin (FPN). FPN is the only iron-exporting protein localized in the cell membrane; it was independently discovered by three different groups [7–9]. The FPN structure has not been completely defined; it is characterized by 9-12 transmembrane domains (TMs), organized into two six-helix halves, which are connected by a large cytoplasmic loop between the 6^th^ and the 7^th^ domain [10, 11]. Furthermore, whether the functional form of FPN is monomeric or dimeric remains an open question. Genetic and biochemical evidences support the dimeric form [12]. However, different groups reported that FPN is a monomer, and that, in this form, it is able to bind HAMP [13, 14]. Regulation of FPN occurs at multiple levels, transcriptional, posttranscriptional, and posttranslational. FPN expression is regulated at the transcriptional level by hypoxia inducible factor-2alpha (HIF2*α*) in response to hypoxia and inflammation; moreover, it is induced by iron heme and other metals. Posttranscriptionally, FPN synthesis is regulated by iron regulatory proteins (IRPs), which bind to an iron responsive element (IRE) located in its 5′UTR. In addition, posttranslational regulation of FPN is mediated by HAMP. HAMP binds FPN and triggers its internalization, ubiquitination, and subsequent lysosomal degradation [10, 11].

At systemic level, circulating HAMP is synthesized by the liver, where it is induced in iron overloading conditions and is inhibited by iron deficiency due to anemia, hypoxia, ineffective erythropoiesis, and inflammation [10, 11]. HAMP is also expressed in the heart, brain, kidney, and placenta [15]; in these tissues, its role is less defined, but it is likely involved in iron handling. HAMP expression is regulated by different members of the TGF-*β* superfamily, including BMP (bone morphogenetic protein) receptors, associated BMP ligands, and the cytoplasmic SMAD transcription factor [16]. Moreover, it is been shown that Atoh8 (atonal bHLH transcription factor 8) contributes to hepcidin regulation in response to iron levels by interacting with Id1 proteins [17]. Cardiac expression of HAMP is induced in response to hypoxia and inflammation [18]. Upregulation of HAMP occurs in heart ischemia [19], while its downregulation was described in a mouse model of dilated cardiomyopathy [20]. Moreover, Hsieh et al. [21] showed that apoptosis was induced by the knockdown of HAMP by siRNA in human cardiomyocytes treated with ferrous iron. Anomalies of HAMP-FPN axis affect the heart functionality. Mouse cardiac FPN knockouts show dilated cardiomyopathy and iron deposits in cardiomyocytes [22]. In addition, it has been shown that HAMP knockout at the cardiac level leads to an increase in cardiac FPN; moreover, HAMP loss or HAMP unresponsiveness is associated to cardiac hypertrophy and apoptosis [23]. In addition, heart autoptic tissue of FRDA patients revealed macrophagic inflammatory infiltrate with high levels of HAMP and iron deposits [24]. To the mechanisms of iron uptake, transport, storage, and export mentioned above, one should add those that regulate the utilization of iron and its correct subcellular distribution. Noteworthy, a still underestimated subcellular compartment involved in iron trafficking is the nucleus, where iron-sulfur clusters associated with DNA repair enzymes [25] and transcription factors have been described [26]. Iron transporters and storage proteins, such as the divalent metal transporter 1 (DMT1), lactoferrin, and ferritin, are associated to the nucleus [27].

The deregulation of iron compartmentalization is very often associated with neurodegenerative pathologies such as Friedrich's ataxia, a progressive neurodegenerative disorder characterized by degeneration of central and peripheral nervous systems and associated with hypertrophic cardiomyopathy and iron deposits [28]. Cardiomyopathy and subsequent cardiac failure is the most common cause of death in FRDA patients [29], where expanded GAA repeats in intron 1 of the frataxin gene (FXN) cause its partial deficit [30]. Physiological functions of frataxin involve iron binding and storage, biogenesis of heme and iron-sulfur clusters, and iron sensing; data suggest that further—still undetermined—functions are present. Frataxin depletion results in mitochondrial dysfunction, mitochondrial iron accumulation, and ROS production [5, 31]. In this context, the complex relationship between the mitochondrial aberrations, iron imbalance and frataxin dysfunction, has contributed to the difficulty of deciphering the molecular mechanisms underlying the iron homeostasis imbalance and consequently of identifying effective therapeutic molecules to mitigate the cardiac hypertrophy. Moreover, the lack of a model that can recapitulate the phenotypic and genotypic characteristics of FRDA contributes to the poor knowledge of the underlying mechanisms of this disease.

Aim of the present study is to generate and characterize iPSC-derived cardiomyocytes as a cellular model to explore the HAMP-FPN axis and investigate the iron homeostasis in FRDA cardiac phenotype. Differentiation of iPSC-derived cardiomyocytes was monitored by cardiac gene analysis with real-time PCR and evaluation of cardiac proteins by cytofluorimetric and immunofluorescence methods.

## 2. Material and Methods

### 2.1. Human-Induced Pluripotent Stem Cells (hiPSCs)

Human iPSCs derived from a healthy subject and from a FRDA patient were obtained from the NIGMS Human Genetic Cell Repository at the Coriell Institute for Medical Research: (GM 23280∗A and GM23404∗B, respectively) and generated through fibroblast reprogramming according to the Yamanaka method [32].  Table S1 reports genotypic and phenotypic features of the subjects.

### 2.2. Cardiomyocyte Derivation from hiPSCs

Differentiation of hiPSCs in cardiomyocytes was performed according to the GiWi method by Lian et al. [33]. The detailed protocol and the timeline of cardiac differentiation are reported in Figure S1.

### 2.3. Flow Cytometric Analysis

For cytometric analysis, wells were washed with PBS 1x and cardiomyocytes were dissociated with trypsin-EDTA 0.25% and then fixed in 1% paraformaldehyde for 20 min at room temperature and 90% cold methanol for 15 min. Cells (0.5 × 10^6^) were centrifuged and the pellet incubated with primary anti-troponin T (TNNT2) antibody (Thermo Fisher Scientific, Waltham, MD) overnight at 4°C in a buffer containing 5% BSA and 1% Triton X-100 in PBS. Secondary antibody (Alexa Fluor 488 Goat anti-mouse IgG1) was added, and the samples were incubated for 30 min at room temperature, after which the nuclei were stained with DAPI. Samples were acquired using a FACSCalibur instrument and analyzed with the CellQuest software (Becton Dickinson, Italy). The primary and secondary antibody and the dilutions used are listed in Table S2.

### 2.4. Immunostaining Analysis

Cardiomyocytes were washed with PBS 1x and were dissociated with trypsin-EDTA 0.25% and then seeded on 0.1% gelatin-coated coversplis at 1 × 10^5^cells/mL. After two days, the cells were fixed in 4% paraformaldehyde for 15 min at room temperature. The primary and secondary antibody and the dilutions used are listed in Table S2. Cardiomyocytes were incubated overnight at 4°C with the primary antibody in a buffer containing 5% nonfat dry milk and 0.4% Triton X-100 in PBS. Cells were washed three times for 5 min in PBS 1x. Subsequently, the cardiomyocytes were incubated with the secondary antibody for 20 min at room temperature, followed by nuclear staining with mounting medium containing DAPI (Santa Cruz Biotechnology, DBA, Italy). Images of cardiomyocytes were obtained using a fluorescence microscope (Leica DMLB Fluo MS15062).

### 2.5. RNA Isolation and cDNA Synthesis

Total RNA was extracted with TRIzol™ reagent (Invitrogen, Milan, Italy) following the manufacturer's instructions. RNA quality was measured by evaluation of 28S and 18S rRNA band sharpness after denaturing electrophoresis. RNA purity and concentration were assessed by spectrophotometer evaluation (Ultrospec 3000, Pharmacia Biotech, Cambridge, UK) at 230, 260, and 280 nm. Reverse transcription (800 ng of RNA template) was performed in a final volume of 20 *μ*L using the iScript cDNA Synthesis Kit (Bio-Rad, Hercules, CA) following the manufacturer's instructions. The cDNA thus obtained was stored at -20°C and used for qRT-PCR analysis.

### 2.6. Quantitative RT-PCR Analysis

Quantitative RT-PCR was performed according to Abruzzo et al. [34] in a Bio-Rad CFX96 real-time thermal cycler using the SsoAdvanced™ SYBR® Green Supermix (Bio-Rad Laboratories, Hercules, CA). The primer sequences for target and housekeeping genes (*β*-actin, GAPDH) are listed in Table S3. Primers were designed with *PRIMER3* and *AMPLIFY* software and, whenever possible, were designed as to span an exon-exon junction. All primers were purchased from Sigma-Aldrich (St. Louis, MO). Data were analyzed with the software CFX Manager software (Bio-Rad Laboratories, Hercules, CA), by using the 2^−ΔΔCT^ method [35]; data were normalized with the housekeeping genes *β*-actin and GAPDH; primer efficiency in the real-time PCR reaction was between 95% and 105% [36].

### 2.7. Bioinformatic Analysis of Ferroportin Protein Sequence

In order to evaluate whether a nuclear localization signal (NLS) is present in FPN, its protein sequence was analyzed by cNLS Mapper, a freely available software (http://nls-mapper.iab.keio.ac.jp/cgi-bin/NLS_Mapper_form.cgi). cNLS is a bioinformatic tool useful to predict the NLS specific for the *αβ* importin pathway; it yields the NLS scores (levels of NLS activities). Four NLS profiles are calculated: class 1/2, class 3, class 4, and bipartite NLSs. cNLS Mapper extracts putative NLS sequences with a score equal to or more than the selected cutoff score. Each amino acid residue at each position within an NLS class yields a score that sums up in order to characterize the entire NLS activity. Higher scores (8, 9, or 10) indicate the exclusive localization in the nucleus; scores 7 or 8 indicate a partial localization in the nucleus; scores 3, 4, or 5 suggest that the protein is localized both in the nucleus and in the cytoplasm. Scores 1 or 2 define the exclusive localization in the cytoplasm [37].

### 2.8. Confocal Analysis of Ferroportin

Confocal microscopy was used to study the presence of the FPN in the nuclei of iPSC-derived cardiomyocytes. Sections were scanned with a Nikon Ti-E fluorescence microscope coupled to an A1R confocal system and the NIS-Elements AR 3.2 software. A diode laser system with 405 wavelength output, air-cooled argon-ion laser system with 488 wavelength output, and yellow diode-pumped solid-state laser system with 561 wavelength output were used. Images were acquired with oil immersion 60x with an optical resolution of 0.18 micron, 2x scanner zoom, and 1024 × 1024 pixel resolution. All the *z* stacks were collected in compliance with optical section separation (*z* interval) values suggested by the NIS-Elements AR 3.2 software. Three random fields per sample were acquired, containing at least 10 cells per sample. Stacks were 0.850 *μ*m for a total of 14 images. 3D images were analyzed by the Imaris software (Bitplane, Concord, MA). The algorithm of the software is able to detect the nuclei, marked by DAPI, and create an isosurface on the blue fluorescence. Then, the green fluorescence, corresponding to the total FPN signal, was quantified only inside the nuclei isosurfaces. In addition, the volume of each nucleus was measured and analyzed.

### 2.9. Western Blot Analysis

Cardiomyocytes were lysed in 50 mM Tris-HCl (pH 8.0), 150 mM NaCl, NP-40 1%, and protease inhibitor mix (Roche, Sigma-Aldrich, Saint Louis, MO). Protein concentration was determined using Bradford protein assay (Bio-Rad Laboratories, Hercules, CA). 45 *μ*g of protein samples was solubilized in Laemmli buffer 4x (200 mM Tris-HCl, pH 6.8, 5% SDS, 25% glycerol, 0.04% bromophenol blue, and 5% beta-mercaptoethanol) for 1 h in ice bath. Precast gradient gels (Mini-PROTEAN TGX stain-free protein gel, 4-15% polyacrylamide) (Bio-Rad Laboratories, Hercules, CA) were used. Mini-PROTEAN TGX gels contain trihalo compounds, which, in the presence of UV light, react with tryptophan residues producing fluorescence, proportional to the total protein amount of the sample. Gels were electroblotted onto nitrocellulose membranes (pore sizes: 0.45 *μ*m). Membranes were exposed to UV light in order to visualize the protein band integrity and the efficiency of transfer. After blocking in Tris-buffered saline (TBS 1x) containing 0.1% Tween-20 (TBS-T), nonfat milk 5%, and 1% BSA for 1 h at room temperature, membranes were probed overnight at 4°C with the primary antibodies and then washed three times with TBS-T and incubated with rabbit-IgG HRP-conjugated secondary antibody, dissolved in blocking buffer for 1 hour at room temperature. Details about primary and secondary antibodies used are listed in Table S2. Finally, membranes were incubated with ECL chemiluminescent reagent (Western Bright ECL HRP substrate, Advansta, CA, USA) and exposed to an X-ray film (Aurogene s.r.l., Rome, Italy). Densitometric analysis was performed by means of Bio-Rad Gel Doc 2000. Density of specific protein bands was normalized to the *β*-actin band.

### 2.10. Statistical Analysis

For quantitative RT-PCR, statistical analysis was performed by the CFX Manager software (Bio-Rad Laboratories, Hercules, CA) and qbase plus (http://www.biogazelle.com/). For Western blot, the statistical analysis was performed by Student's *t*-test. A value of *p* < 0.05 was considered statistically significant.

## 3. Results

### 3.1. Cardiomyocyte Differentiation from hiPSCs

The differentiation of cardiomyocytes (CMs) from iPSCs was successfully repeated four times. Movies SM1 and SM2, reported in the Supplementary Materials, show beating CMs from both control and FRDA cultures. Notably, some of the beating areas found in cardiomyocyte cultures derived from FRDA iPSCs were unsynchronized, in contrast with the remarkable synchronization of control cardiomyocyte cultures.

### 3.2. Cardiomyocyte Characterization

**Figure 1:**
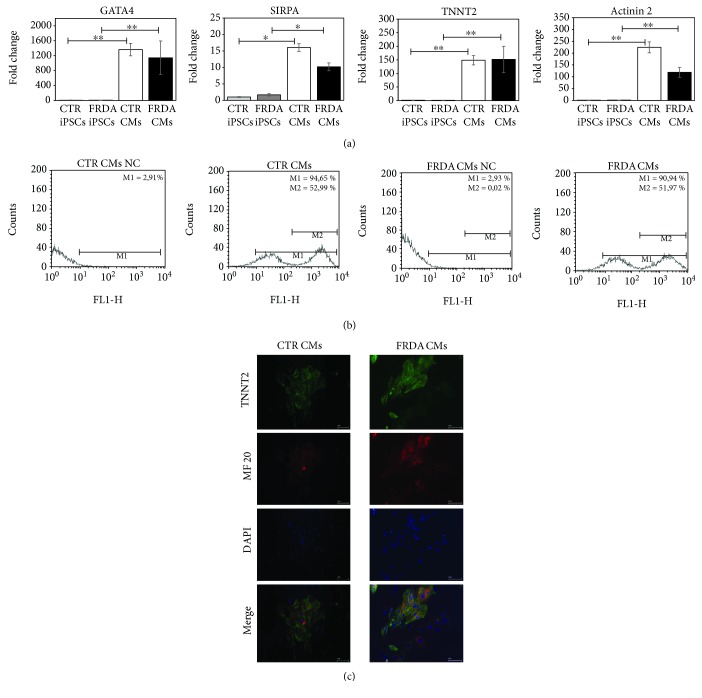
Characterization of CTR and FRDA iPSCs and iPSC-derived CMs. (a) Gene expression of four specific cardiac genes, GATA4, SIRPA, TNNT2, and actinin 2 characterized by qRT-PCR. Data were normalized with two housekeeping genes, *β*-actin and GAPDH; for each gene, the normalized expression value of CTR iPSCs was set to 1, and all other gene expression data were reported to that sample. PCR was run in triplicate; data are from four independent differentiation experiments and are expressed as mean ± SEM. ^∗^ *p* < 0.05; ^∗∗^ *p* < 0.01. (b) A representative flow cytometer analysis of CTR and FRDA CMs stained with TNNT2. NC negative control was stained with secondary antibody only; M1: percentage of TNNT2-positive CMs; M2: percentage of CMs highly positive to TNNT2. (c) A representative immunofluorescence image of CTR and FRDA CMs. TNNT2 is stained in green; heavy myosin chain (MF 20) is stained in red; the nuclei are stained with DAPI (blue). Scale bar 50 *μ*m.

The characterization of cardiomyocyte differentiation was obtained by evaluating the synthesis of four heart-specific genes: GATA4, a transcription factor specific for the cardiac lineage; SIRPA, a nonreceptorial tyrosine protein-phosphatase, exclusively expressed on the surface of hiPSC-derived cardiomyocytes [38]; and TNNT2 and actinin 2, two cardiac structural proteins. Messenger RNAs from control and FRDA CMs and iPSCs were compared by qRT-PCR and results are shown in Figure 1(a) as the average of four independent differentiation procedures. Cardiac-specific genes were significantly upregulated in CMs with respect to iPSCs in both control and FRDA samples.

Moreover, TNNT2 protein was evaluated by FACS analysis in CMs (Figure 1(b)). FACS analysis showed that the efficiency of CM differentiation from iPSCs was 80-90%.

Finally, the immunofluorescence analysis demonstrated that TNNT2 costained with the heavy myosin chain (MF20) proteins (Figure 1(c)) in both control and FRDA CMs. TNNT2 fluorescence suggests that both control and FRDA CMs display the typical sarcomeric organization.

### 3.3. Maintenance of the FRDA-Like Phenotype in iPSCs and CMs

**Figure 2:**
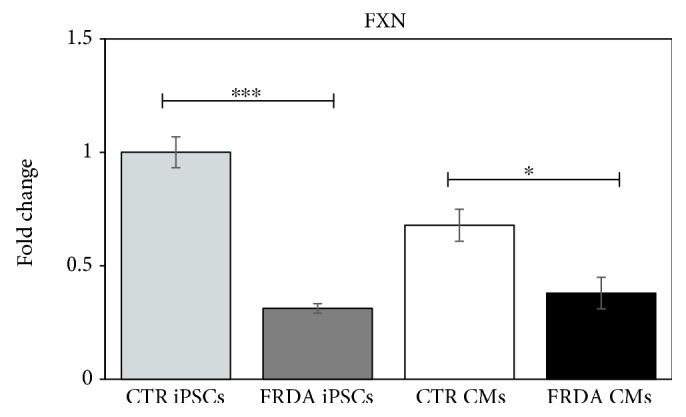
Evaluation of the FRDA-like phenotype in iPSCs and CMs. qRT-PCR of FXN gene expression in both CTR and FRDA iPSCs and CMs. Data were normalized with two housekeeping genes, *β*-actin and GAPDH; the normalized FXN expression value of CTR iPSCs was set to 1, and all other gene expression data were reported to that sample. Data are from four independent differentiation experiments and are expressed as mean ± SEM. ^∗^ *p* < 0.05; ^∗∗∗^ *p* < 0.001.

To determine whether FRDA iPSCs and CMs maintained the FRDA-like phenotype, the gene expression of FXN was analyzed. As expected, the expression of FXN gene in FRDA iPSCs was about 30% of control iPSCs (Figure 2). Moreover, cardiac differentiation did not alter the pathological phenotype; the expression of FXN in FRDA CMs was about 55% with respect to control. An average of four independent experiments is shown.

### 3.4. TFRC Gene Expression Is Upregulated in FRDA CMs

**Figure 3:**
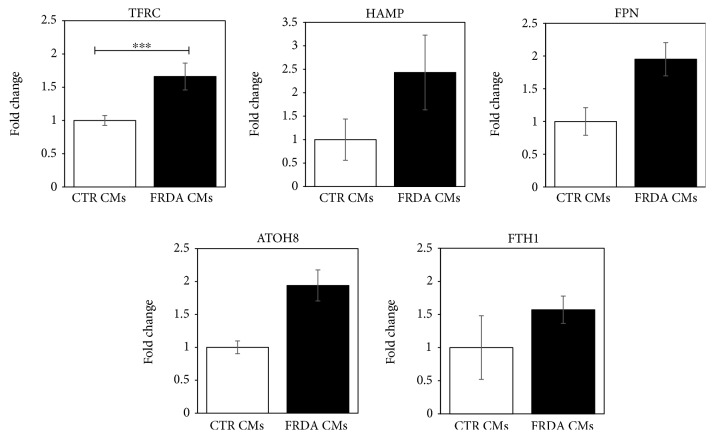
Gene expression of proteins involved in iron homeostasis in CTR and FRDA CMs. Expression levels of TFRC, HAMP, FPN, ATOH8, and FTH1 genes in CTR and FRDA CMs evaluated by qRT-PCR. Data were normalized with two housekeeping genes, *β*-actin and GAPDH; for each gene, the normalized expression value of CTR CMs was set to 1, and all other gene expression data were reported to that sample. Data are from four independent differentiation experiments and are expressed as mean ± SEM. ^∗∗∗^ *p* < 0.001.

The mRNA abundance of the key iron homeostasis-related genes was assessed in control and FRDA CMs by qRT-PCR. Data are shown in Figure 3 as the average of four independent differentiation procedures. The expression of transferrin receptor (TFRC) was significantly increased in iPSC-CMs FRDA. Moreover, also the mRNA levels of HAMP, FPN, and ATOH8, a transcription factor involved in HAMP regulation, showed a trend to increase. No relevant difference was evidenced in the expression of FTH1 gene.

### 3.5. Protein Expression of HAMP and FPN Is Increased in FRDA CMs

**Figure 4:**
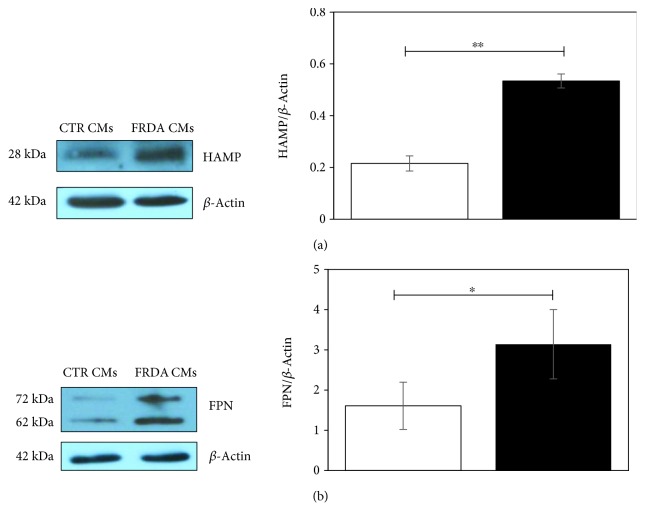
Protein expression of HAMP and FPN. (a) A representative Western blot image of HAMP expression. The band densities were analyzed by densitometry and normalized to *β*-actin. Histogram showing means ± Std Dev of HAMP/*β*-actin. (b) A representative Western blot image of FPN expression. The band densities were analyzed by densitometry and normalized to *β*-actin. Histogram showing means ± Std Dev of FPN/*β*-actin. Data are from three independent differentiation experiments. ^∗^ *p* < 0.05; ^∗∗^ *p* < 0.01.

To validate gene expression data, the protein amount of HAMP and FPN by Western blot was evaluated in whole lysates of both CTR and FRDA CMs. A representative image and the densitometric analysis of the bands are shown in Figures 4(a) and 4(b); the average of three independent differentiation experiments is reported. A significant increase of HAMP and FPN (about 2.5 and 2.0 times, respectively) was found in FRDA CMs compared to controls. It should be noted that FPN antibody detects two bands, at about 72 kDa and 62 kDa, respectively, both of which were more intense in FRDA CMs with respect to controls. Ross et al. [39] described two FPN bands, at ~65 and ≈55 kDa, respectively, in T-REx™/FPN-V5 cells and demonstrated that the heavier isoform was glycosylated.

### 3.6. FPN Nuclear Localization in iPSC-Derived CMs

**Figure 5:**
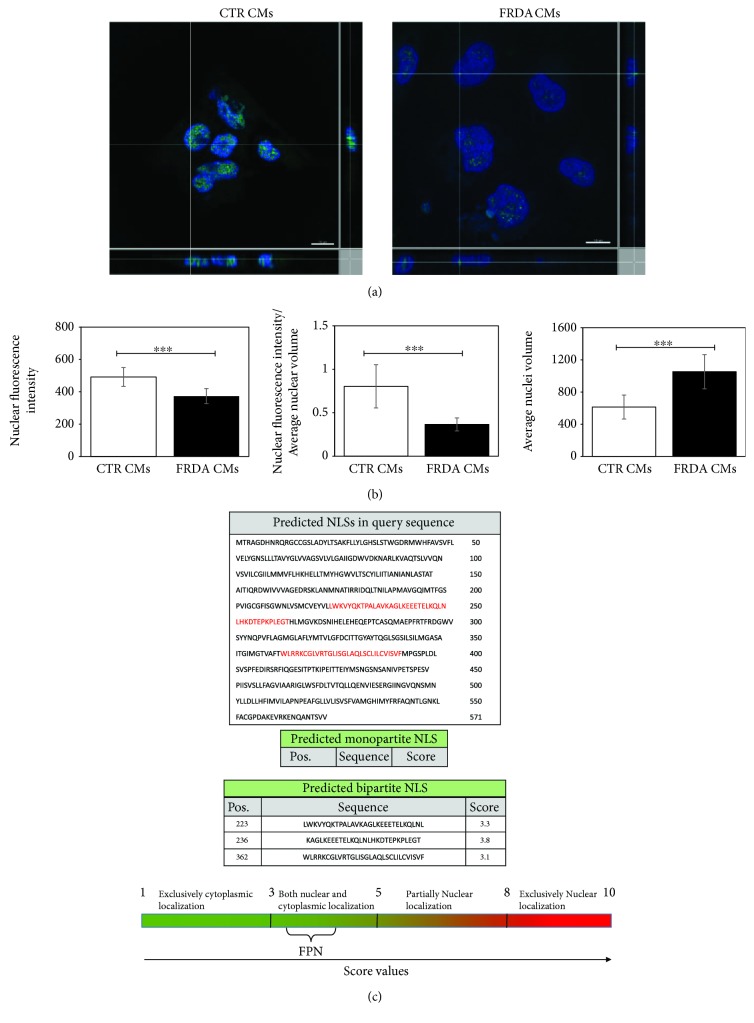
FPN nuclear localization in iPSC-derived CMs. (a) A representative confocal microscopy 3D image projection of FITC-stained FPN (green) protein expression in both CTR and FRDA CMs; the nuclei are counterstained with DAPI (blue). Scale bar equal 10 *μ*m. (b) (i) Quantitative analysis of nuclear FITC fluorescence intensity in both CTR and FRDA CMs, (ii) quantitative analysis of nuclear FITC fluorescence intensity normalized to the average nuclear volume in both CTR and FRDA CMs, and (iii) average nuclear volume in both CTR and FRDA CMs; ^∗∗∗^ *p* < 0.001. (c) Bioinformatic analysis of FPN (NP_055400), using cNLS Mapper, predicted three bipartite NLS with a score value >3 indicating that the protein localizes both in the nucleus and in the cytoplasm.

FPN protein localization and expression was assessed by immunostaining using confocal microscopy.  Figure 5(a) clearly demonstrates a nuclear localization of FPN in both CTR and FRDA CMs. Moreover, the analysis of FITC fluorescence intensity (Figure 5(b)) showed that (i) the amount of FPN was significantly lower in the nuclei from FRDA CMs compared to controls and (ii) decrease was stronger when normalized to the volume of the nucleus, which (iii) is greater in FRDA CMs than in control. Since the nuclear localization of FPN had not been previously described, we used the cNLS Mapper software to identify, if present, nuclear localization signals (NLS) in the FPN protein sequence (NP_055400). This analysis revealed three predicted bipartite nuclear localization sequences: a sequence of 29 aa in 223 position (LWKVYQKTPALAVKAGLKEEETELKQLNL) with 3.3 score, a sequence of 32 aa (WLRRKCGLVRTGLISGLAQLSCLILCVISVF) in 362 position with 3.1 score, and finally a sequence of 29 aa (KAGLKEEETELKQLNLHKDTEPKPLEGTH) in 236 position with 3.8 score (Figure 5(c)). The NLS score analysis suggests that FPN can be localized in both the nucleus and cytoplasm.

## 4. Discussion

In the present study, we describe some relevant features of iron homeostasis in iPSC-derived cardiomyocytes from one healthy control and one FRDA-affected patient. Cardiomyocytes were shown to fully express cardiac differentiation markers. Both FRDA iPSCs and CMs maintained the pathological phenotype, characterized by low levels of frataxin mRNA; however, frataxin expression in FRDA CMs was not as reduced as in iPSCs relative to control CMs. A similar result was reported by Hick et al. [40], who ascribed such difference to the reduced number of GAA repeats in the CM beating areas with respect to iPSCs of the same subject. Spontaneously beating areas were observed at 9-12 days from the start of the induction procedure, but FRDA beating areas were desynchronized, in agreement with similar results reported by Hick et al. [40].

Several studies pointed out the importance of iron homeostasis in the cardiac tissue; however, to date, the local regulation of iron in cardiomyocytes has not been fully characterized. In particular, the HAMP-FPN axis seems to be crucial for heart function [15, 22, 23]. Cardiac iron dysregulation was described in several disorders including FRDA, which is characterized by iron maldistribution within subcellular compartments, leading to mitochondrial iron accumulation and cytosolic iron depletion [5, 28, 41]. In the present study, cardiomyocytes were derived from CTR and FRDA human iPSCs in order to study some features of iron homeostasis, focusing the attention to the HAMP-FPN axis. This cellular model was already exploited by Lee et al. [42] to study the gene and protein expression of a group of iron-handling proteins in FRDA. To our knowledge, iron distribution within the cell compartments has not yet been evaluated in the iPSC-derived CM cellular model. The lack of such evaluation is a limitation of the present study, but this issue will be addressed in future research.

In the present study, a number of iron homeostasis-related genes and proteins were found to be dysregulated in FRDA CMs. Elevated HAMP expression has been already described in FRDA autoptic heart tissues [24], but the authors attributed HAMP overexpression to macrophagic inflammatory infiltrate. On the contrary, our results suggest that the increase in HAMP content is due to its overexpression in FRDA CMs.

In turn, HAMP downregulates the cellular amount of FPN, causing its lysosomal degradation [10, 11]. At variance with the expected decrease of FPN levels, HAMP upregulation in FRDA CMs was not accompanied by a decrease in FPN, rather a significant increase of FPN (protein) was observed.

In a different context, both FPN and HAMP were found to be significantly decreased in brain tissue of Alzheimer's disease patients, where oxidative stress is known to occur [43].

Our data show that the upregulation of HAMP and FPN in FRDA CMs is accompanied by the upregulation of TFRC. Huang et al. [44] found a decrease of FPN and an increase of TFRC in the heart of a conditional frataxin knockout (mutant) mice. It is difficult to compare our in vitro data with this study, which makes use of an animal model, where a complex interplay between local and systemic iron homeostasis takes place, and where frataxin expression is almost completely abolished. It is our opinion that our observations may reflect a dysregulation of these iron-handling proteins in FRDA CMs. Since it is known that oxidative stress affects FRDA neuronal and cardiac cells [45], it is possible that FPN loses its responsiveness to HAMP downregulation owing to the oxidation of key cysteine residues located in the HAMP- and FPN-interacting sites [46–48]. It can be envisioned that these cysteine residues are not oxidized in a “normal” low iron context, where oxidative stress is not present and HAMP downregulates FPN in order to avoid a further iron depletion. However, in the cytoplasm of FRDA cardiomyocytes, an unusual concurrence of low iron concentration AND oxidative stress is present, which would favor the inability of HAMP to downregulate FPN and to avoid iron leakage. Obviously, this is only one out of several other hypotheses, and further investigations need to be carried out for its validation or disproval.

The presence of FPN in the nuclei of CTR and FRDA CMs was demonstrated in this study for the first time. This finding suggests that FPN, traditionally considered as a transmembrane protein, could play a role in the maintenance of nuclear iron homeostasis. Henle et al. [49] described specific iron-binding sites on DNA. Moreover, other proteins involved in iron cellular trafficking, such as the divalent metal transporter 1 (DMT1), lactoferrin, and ferritin [27], have been found in the nuclear compartment. It is possible that the nuclear localization of FPN underscores a protective role from excess free iron in the nuclear compartment. Surprisingly, we found a lesser amount of nuclear FPN in FRDA cardiomyocytes with respect to controls, a result that seems to support the finding of DNA oxidative stress markers (8-OXO-dg) in FRDA patients [50]. On the other hand, the fact that FPN is less abundant in the nuclei of FRDA CMs than in controls is not necessarily in contradiction with the increase in both mRNA and protein FPN in FRDA cardiomyocytes, since Western blots were carried out in whole cell lysates, which include cytoplasmic FPN. A limitation of the present study is the lack of quantification of cytoplasmic FPN, either by confocal microscopy (which would require the delimitation of cell boundary by a specific staining) or by subcellular fractioning. This will be addressed by future studies.

The regulation of FPN distribution among the different subcellular compartments is a relevant issue, not yet afforded by researchers; in fact, the presence of 9-12 transmembrane domains supports its localization in the plasma membrane and its role of iron exporter and makes less likely a cytoplasmic and a nuclear localization. Thus, the nuclear localization of FPN we document here needs to be discussed not only in terms of novel FPN function(s), but also within the context of membrane and organelle trafficking and of final destination of proteins characterized by hydrophobic domains calling for a plasma membrane localization.

Finally, the presence of the enlarged nuclei, which was described here to occur in FRDA cardiomyocytes, has been reported in other cardiac pathologies, such as in hypertrophic and in dilated cardiomyopathy [51]. These morphological data need to be further investigated.

## 5. Conclusions

Cardiomyocytes derived from iPSCs retained the FRDA-like phenotype. Important alterations in the expression of HAMP and FPN, two proteins that play pivotal roles in cardiac iron homeostasis, are described here. Moreover, a novel nuclear localization of FPN in cardiomyocytes is reported, which suggests a potential new physiological function of this protein. These findings may have important implications in the understanding of cardiac iron homeostasis in both physiological and pathological conditions, such as FRDA. In particular, FRDA cardiomyocytes appear to be unable to exploit HAMP-operated regulation of FPN, which might be one of the reasons why iron distribution within the cell is impaired, thus leading to increase in the free iron pool. This, together with the defective assembly of mitochondrial proteins, would lead to chronic oxidative stress in FRDA cardiac cells. As pointed out by many authors [52–54], oxidative stress does not only lead to cell damage and apoptosis but also plays a role in adverse remodeling and contractile dysfunctions, as seen in FRDA patients.

## Data Availability

The data used to support the findings of this study are available from the corresponding author upon request.

## Abbreviations

ATOH8: Atonal BHLH transcription factor 8
BMPs: Bone morphogenetic proteins
CMs: Cardiomyocytes
DMT1: Divalent metal transporter 1
FACS: Flow cytometry
FPN: Ferroportin
FRDA: Friedreich's ataxia
FTH1: Ferritin heavy chain 1
FXN: Frataxin gene
GAPDH: Glyceraldehyde-3-phosphate dehydrogenase
HAMP: Hepcidin
HH: Hereditary hemochromatosis
hiPSCs: Human-induced pluripotent stem cells
IF: Immunofluorescence
IREs: Iron regulatory elements
IRPs: Iron regulatory proteins
ISC: Iron-sulfur cluster
LIP: Labile iron pool
MF 20: Myosin heavy chain
NLS: Nuclear localization signal
ROS: Reactive oxygen species
TFRC: Transferrin receptor 1
TNNT2: Cardiac troponin T
TMs: Transmembrane domains.

## Supplementary Materials

Supplementary 1 Table S1: phenotypic data of control subject and FRDA patient from which iPSCs were generated by Coriell Institute Repositories. Table S2: list of primary and secondary antibodies used in cytofluorimetry (FACS), immunofluorescence (IF), and Western blot (WB) analysis. Table S3: primer sequences and amplicon length of the genes analyzed by real-time PCR in iPSC-derived cardiomyocytes. *β*-Actin and GADPH were used as housekeeping genes.

Supplementary 2 Figure S1: timeline of iPSC-derived cardiomyocyte differentiation protocol.

Supplementary 3 SM1: CTR cardiomyocytes.

Supplementary 4 SM2: FRDA cardiomyocytes.
