# Supplementary material for: New Insights into the Hepcidin-Ferroportin Axis and Iron Homeostasis in iPSC-Derived Cardiomyocytes from Friedreich's Ataxia Patient
Source: Oxid Med Cell Longev. 2019 Mar 27;2019:7623023. doi: 10.1155/2019/7623023 (PMC6458886; doi:10.1155/2019/7623023)
Supplement: Supplementary 1 — Table S1: phenotypic data of control subject and FRDA patient from which iPSCs were generated by Coriell Institute Repositories. Table S2: list of primary and secondary antibodies used in cytofluorimetry (FACS), immunofluorescence (IF), and Western blot (WB) analysis. Table S3: primer sequences and amplicon length of the genes analyzed by real-time PCR in iPSC-derived cardiomyocytes. β-Actin and GADPH were used as housekeeping genes. [file 7623023.f1.pdf]

SUPPLEMENTARY MATERIALS AND METHODS

1. Cardiomyocytes derivation from hiPSCs

The iPSC colonies were grown in 6-well dishes on irradiated mouse embryonic fibroblasts (GlobalStem, Rockville, MD) in DMEM/F-12 with 20% KnockOut serum replacement, 1 mM glutamine, 1% non-essential amino acids, 1% penicillin/streptomycin, 0.1 mM Beta-mercaptoethanol (Life Tecnologies, USA), 20 or 30 ng/mL basic FGF (Stemgent, San Diego, CA) and were passaged manually every 7 or 8 days. When iPSC colonies reached confluency, they were dissociated with Versene (Life Tecnologies, USA) and were growth on Matrigel-coated plates in mTeSr1 medium (STEM CELLS, Tecnologies,USA) with 5µM ROCK inhibitor (Y27632, Miltenyi Biotec, Germany). Following confluency in the feeder-free condition, iPSC colonies were detached with Accutase (Life Tecnologies, USA) and then replated on a 12-well Matrigel-coated dish in mTeSr1 medium with 5µM ROCK inhibitor (Y27632) (Miltenyi Biotec, Germany) at different cell densities: 0.5, 0.75 or 1 milion cells/mL for four days. Induction was performed with 12 µM CHIR99021 (Selleckchem, USA) in RPMI medium supplemented with B27-insulin (Life Tecnologies, USA). Twenty-four hours later, the medium was substituted with RPMI with B27-insulin. The following day, the medium was substituted with 2 mL of a medium made up of 1 mL of the medium collected from the well and 1 mL of fresh RPMI with B27-insulin, to which 5 µM IWP2 (Tocris, Bioscience, USA) was added. This medium was replaced two days later with RPMI with B27-insulin. Two days later, RPMI with B27 was substituted in the wells. First beating areas are expected to form between the 9th and the 12th day from the culture in mTeSr1 medium. Cardiomyocytes with spontaneously beating area were maintained in RPMI/ B27 medium until the 20th day of induction.

SUPPLEMENTARY TABLES

Table S1: phenotypic data of control subject and FRDA patient from which iPSCs were generated by Coriell Institute Repositories.

CODE	Gender	Age (Years)	GAA length repeats	Phenotypic Data
GM23280*A	♀	36		Apparently healthy
GM23404*B	♀	36	330-380	Spinal-cerebral degeneration; myocardiopathy.

Table S2: list of primary and secondary antibodies used in cytofluorimetry (FACS), immunofluorescence (IF) and Western Blot (WB) analysis

**Antibody**	**Species**	**Diluition**
Anti-Hepcidin, Abcam (Ab30760)	Rabbit polyclonal	1:100 for WB<br>1:100 for IF
Anti-Ferroportin/SLC40A1, Novus biologicals (NBP1-21502)	Rabbit polyclonal	1:500 for WB<br>1:500 for IF
Cardiac Troponin T, Thermo Fisher Scientific (ms-295-p1/13-11)	Mouse IgG1	1:200 for FACS and IF
MF 20, DSHB	Mouse IgG2b	1:20 for IF
Alexa 488 Goat, Life Technologies (A-21121)	Mouse IgG1	1:1000 for FACS and IF
Alexa 594 Goat, Life Technologies (A-21145)	Mouse IgG2b	1:1000 for IF
Anti-Rabbit IgG-FITC, Sigma (F7512)	Rabbit IgG	1:200 for IF
Anti-Rabbit IgG-HRP, Thermo Fisher Scientific (31460)	Rabbit IgG	1:10000 for WB

Table S3: primer sequences and amplicon length of the genes analysed by real-time PCR in iPSC-derived cardiomyocytes. β-Actin and GADPH were used as housekeeping genes.

GENE	NCBI RefSeqs*	*LEFT PRIMER*	*RIGHT PRIMER*	A.L. (bp)**
β-Actin	NM_0011013	agacctgtacgccaacacagt	agtacttgcgctcaggagga	140
GAPDH	NM_002046	cctgacctgccgtctagaaa	tgctgtagccaaattcgttg	233
Frataxin (FXN)	NM_000144	tagcagaggaaacgctggac	gatttgcttgtttggcgtct	170
Hepcdin (HAMP)	NM_021175.2	gaccagtggctctgttttcc	cacatcccacactttgatcg	430
ATOH8	NM_032827	caagaagcgcaaggagtgac	gcatcttggagaagaccacga	125
Ferritin (FTH1)	NM_002032.2	ctggccactgacaaaaatga	cagggtgtgcttgtcaaaga	168
Ferroportin (FPN)	NM_014585.5	cgtcattgctgctagaatcg	ccaggatgaccatgatgaaa	300
Transferrin Receptor (TFRC)	NM_001128148.1	aaaatccggtgtaggcacag	ttaaatgcagggacgaaagg	420
Actinin 2	NM_001103.3	ccgcattatgaccctggtag	gatgcagtactgggcctgat	205
GATA4	NM_001308093.1	tggcctgtcatctcactacg	aagaccaggctgttccaaga	192
SIRPA	NM_001040022.1	agcaaaagccatgacctgaa	cctgggctttcttctgtctg	184
Troponin T2, cardiac type (TNNT2)	NM_000364.3	tccgaaacaggatcaacgat	ccatttccaaacaggagctg	190

*NCBI RefSqs= NBCI Reference Sequences

**A.L. (bp) =Amplicon length (base pair)

SUPPLEMENTARY FIGURES

Figure S1: timeline of iPSC-derived cardiomyocyte differentiation protocol

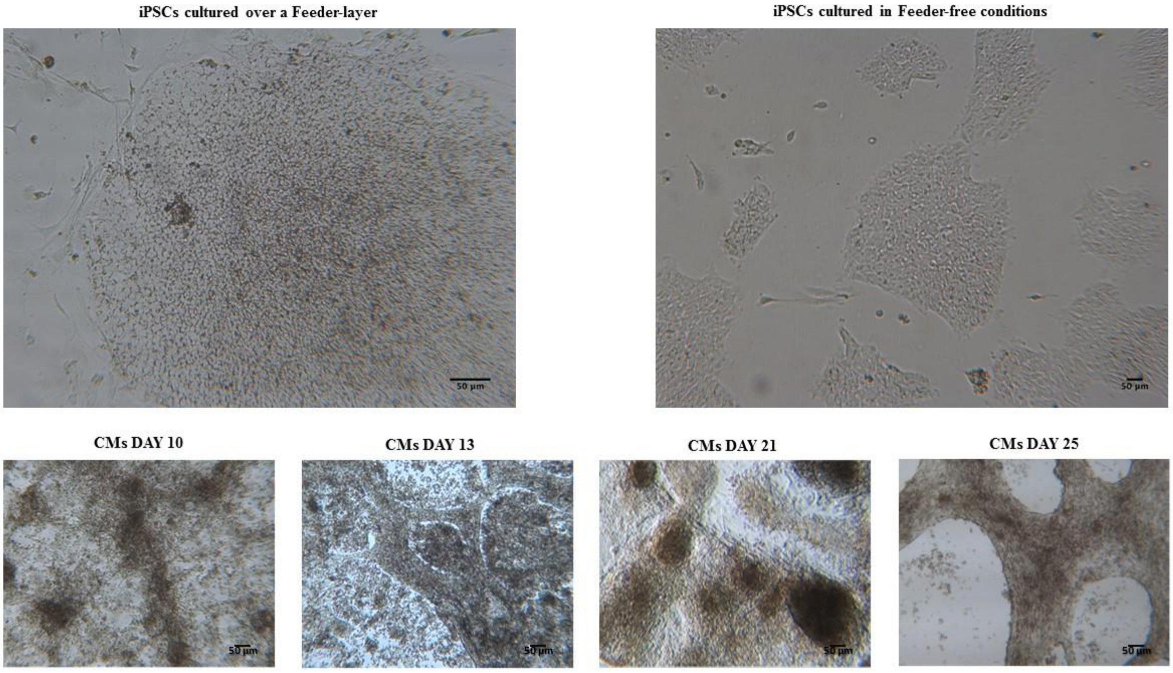

SUPPLEMENTARY VIDEO CAPTIONS

SM1: CTR Cardiomyocytes
SM2: FRDA Cardiomyocytes
