# Supplementary material for: New Insights into the Hepcidin-Ferroportin Axis and Iron Homeostasis in iPSC-Derived Cardiomyocytes from Friedreich's Ataxia Patient
Source: Oxid Med Cell Longev. 2019 Mar 27;2019:7623023. doi: 10.1155/2019/7623023 (PMC6458886; doi:10.1155/2019/7623023)
Supplement: Supplementary 2 — Figure S1: timeline of iPSC-derived cardiomyocyte differentiation protocol. [file 7623023.f2.pptx]

## Slide 1
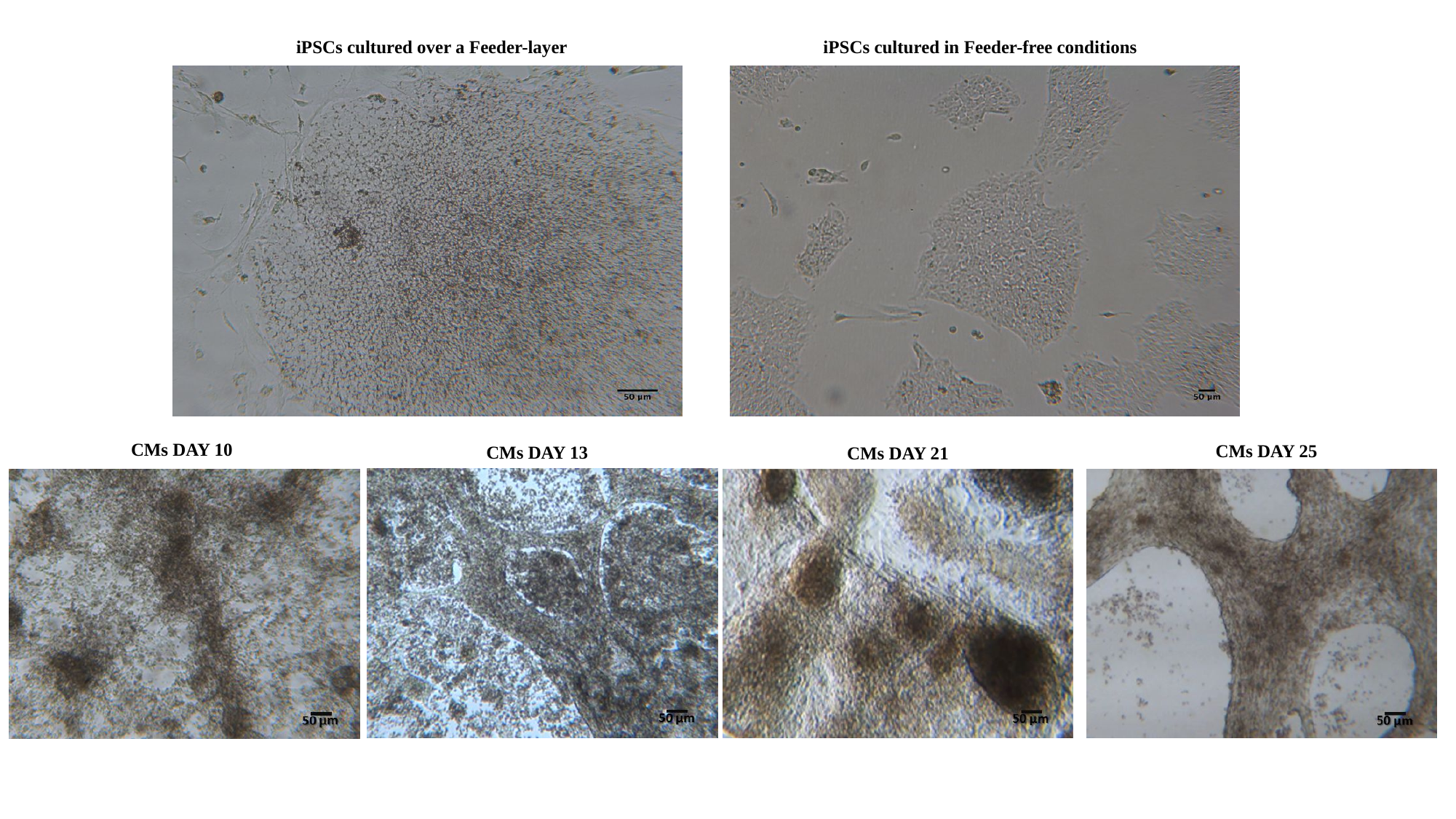

iPSCs cultured in Feeder-free conditions
iPSCs cultured over a Feeder-layer
CMs DAY 10
CMs DAY 25
CMs DAY 13
CMs DAY 21
